# Robust process capability indices C_pm_ and C_pmk_ using Weibull process

**DOI:** 10.1038/s41598-023-44267-4

**Published:** 2023-10-09

**Authors:** Muhammad Kashif, Sami Ullah, Muhammad Aslam, Muhammad Zafer Iqbal

**Affiliations:** 1 Department of Mathematics and Statistics, University of Agriculture, Faisalabad, Pakistan; 2 College of Agriculture, University of Sargodha, Sargodha, Pakistan; 3 Department of Statistics, Faculty of Science, King Abdulaziz University, 21551 Jeddah, Saudi Arabia

**Keywords:** Engineering, Mathematics and computing

## Abstract

Process Capability Indices (PCIs) are very helpful to measure the manufacturing capability and production quality of the products in many manufacturing processes. These PCIs are calculated by using a relationship between process mean and standard deviation, provided that process follows a normal distribution. In case of non-normal processes many researchers recommended the use of robust PCIs by modifying the classical PCIs. In case of robust PCIs most of the work is reported on first- and second-generation PCIs but less work is reported on third generation PCIs. The objective of this work was to evaluate the efficiency of three dispersion measures, namely median absolute deviation (MAD), interquartile range (IQR), and Gini's mean difference (GMD), as a measure of dispersion in third generation PCIs and construct their bootstrap confidence intervals (CIs). The efficacy of these measures is compared with quantile-based PCIs under different asymmetric behaviour of the Weibull process. The results showed that quantile-based PCIs are strongly influenced by high asymmetry and IQR method provides a poor estimator across all sample sizes. On the other hand, the GMD method performed well under low, moderate, and high asymmetry of the Weibull process, but its irregular behavior needs to be addressed carefully. Among all selected four methods MAD-method performed better under low and moderate asymmetric conditions. In case of interval estimation, bias-corrected percentile (BCPB) CIs was recommended for quantile-based PCIs, while percentile (PB) and percentile-t (PTB) CIs were recommended for MAD-based PCIs under all asymmetric conditions. To validate the simulated findings, two real-world datasets were analyzed that supported the simulation results.

## Introduction

The Process Capability Indices (PCIs) are statistical measures used to assess the ability of a process. These indices provide an indication of how well a process meets customer requirements and helpful to identify areas for improvement. The term PCI was first introduced by^1^ and after that there has been extensive research on the usage and implementation of PCIs in various industries and sectors. Researchers and practitioners^2–7^ have explored different aspects of PCIs to enhance their effectiveness and applicability in different contexts. Several indices have been defined but most commonly used are ${C}_{p}, {C}_{pk}, {C}_{pm}$ and ${C}_{pmk}$^2^. defined a supersaturated generalized PCI based on two non-negative parameters $(\lambda ,v)$ for these four indices as

1 $${C}_{p}\left(\lambda ,\nu \right)=\frac{d-\lambda \left|\mu -M\right|}{3\sqrt{{\sigma }^{2}+{\nu (\mu -T)}^{2}}}$$

All four basic PCIs can be generated by putting $\lambda$ and $v$ as $0$ and 1. These indices rely on the assumption that the underlying process data follows a normal distribution. In this case, the PCIs are calculated using the mean and standard deviation of the data^3,7^. However, it's worth noting that there are alternative methods available for estimating PCIs when the normality assumption is violated or when dealing with non-normal data^7^. Various methodological avenues have been investigated for non-normal quality characteristics which can be categorized into different categories (i) Transform non-normal data into normal and use the traditional PCIs. Box-Cox transformation, Johnson transformation system and Clement’s methods using Pearson curves are commonly used transformations to make data normal^8–11^. preferred to compute PCIs from the transformed data (ii) Develop some modified or robust PCIs useful for non-normal data^3,4,10,12–18^. have used some robust measures to compute PCIs. Comparison of different approaches is given in^7,17,19–21^ and references therein. Focusing on the alternate measures to compute PCI under non-normality, the most attractive method was suggested by^12^. The modification of replacing natural interval $\left(6\sigma \right)$ by the width of 99.865th and 0.135th percentile of distribution in ${C}_{p}$ proved to be more reliable for Pearson family of the distribution. The method is simple and attractive for practical and theoretical point of view because it does not require the transformation of the data. Later on, Pearn and Kotz^22^ used Clement’s approach and defined other two indices ${\text{C}}_{\text{pm}}$ and ${\text{C}}_{\text{pmk}}$^23^. modified the Clements idea and used single measure for all cases and defined all four PCIs. It is shown that the three processes, one on-target and the other two off-target, proved that the modified estimator outperforms the original Clements estimators. In another study, Kashif et al.^4^ examine the effectiveness of modified PCIs based on the Pearn-and-Chen quantile method. They discovered that the Gini's mean difference is a more trustworthy indicator of Weibull-based data variability. A very limited application is available for PCIs based on Clements approach for different asymmetric behavior of non-normal distributions^24^. Moreover, the sample standard deviation is based on the assumption of normality and is sensitive to outliers. In case of non-normal data or data contain outliers, the sample standard deviation may not accurately represent the dispersion of the underlying population and alternative estimator of population variability is recommended in the literature^3,4,7,25,26^. Among these alternatives, median absolute deviation (MAD) is one of the robust measures which is less sensitive to extreme values and does not assume a specific distribution. It provides a more robust estimate of the spread of the data and is less affected by outliers. More details on these topics can be seen in^27–36^.

For some non-normal distributions, various modified PCIs based on robust location and dispersion measures have demonstrated promising results. Kashif et al.^3,4^ has presented comparison of first and second generation PCIs by which are based on some robust measures for Weibull distribution.

But the performance of third generation PCIs ${(C}_{pm} \; \&\; {C}_{pmk})$ is yet need to be evaluated for different asymmetric behavior of different non-normal distributions. In this study, the hypothesis is that utilizing robust measures to estimate process variability can yield more accurate estimates for the third generation PCIs in non-normal distribution. For this purpose, Median Absolute Deviation (MAD), Gini’s Mean Difference (GMD) and Inter Quartile Range (IQR) are considered as robust measures that may perform well under non-normality.

Keeping in view the presented problem the present study is planned to evaluate the performance of three robust scale measures: $MAD, \; GMD \;and\; IQR$ in third generation PCIs and compare their performance with quantile-based PCIs using Weibull process. Further to construct bootstrap confidence intervals of processed robust PCIs using different asymmetric behavior of Weibull processes. The rest of the paper is structured as follows: “Material and methods” section explains the third generation PCIs based on the aforementioned robust measures. “Results and discussion” section reports the comparison of the robust third generation PCIs to the PC method, along with their interval estimation.

## Material and methods

### Robust process capability indices

The idea of the use of robust measures in PCIs was introduced by^16^. However, the possible effects on PCIs are somewhat less known. On the other hand, the robust methods have been successfully utilized in the development of control chart theory^25,37–39^. As noted by^39^ some robust measure of variability should be used when using median as measure of central tendency instead of sample mean. Here in this study three robust measures for dispersion, Median Absolute Deviation (MAD), Inter Quartile Range (IQR) and Gini’s Mean Difference (GMD) are considered to derive robust PCIs.

### Quantile based PCI

Suppose that $y$ is a random variable with probability distribution $f\left(x,\theta \right)$, where $\theta$ is a single unknown parameter. Let $[{y}_{1},{y}_{2},\dots .,{y}_{n}]$ be an i.i.d random sample selected from the process having density $f\left(x,\theta \right)$. $\theta ={\left({\theta }_{1},\dots ,{\theta }_{k}\right)}^{\tau }$ is the transpose of the column vector of process parameters. The likelihood and log likelihood function of $\theta$ are given by

2 $$L\left(\theta \right)=\prod_{i=1}^{n}f\left({y}_{i},\theta \right).$$

3 $$l\left(\theta \right)=\sum_{i=1}^{n}lnf\left({y}_{i},\theta \right).$$

respectively. The $\alpha$-quantile of the process distribution is defined implicitly by the function

4 $$\alpha =F\left({\mathbb{Q}}_{\alpha };\theta \right)={\int }_{-\infty }^{{\mathbb{Q}}_{\alpha }}f\left(y,\theta \right)dy .$$

Then the quantile-based PCI superstructure is a function of the population parameter $\theta$. That is

5 $${C}_{Np}\left(\eta ,\kappa ,\theta \right)=\frac{d-\eta \left|{\mathbb{Q}}_{{p}_{2}}(\theta )-m\right|}{3\sqrt{{\left[\frac{{\mathbb{Q}}_{{p}_{3}}(\theta )-{\mathbb{Q}}_{{p}_{1}}(\theta )}{6}\right]}^{2}+\kappa {\left({\mathbb{Q}}_{{p}_{2}}(\theta )-T\right)}^{2}}}$$

Let $\widehat{\theta }={\left({\widehat{\theta }}_{1},{\widehat{\theta }}_{2},\dots ,{\widehat{\theta }}_{k}\right)}^{\tau }$ which maximizes $L\left(\theta \right)$ or $l\left(\theta \right)$, be the MLE of $\theta$. The maximum likelihood estimator of quantile ${\mathbb{Q}}_{\alpha }$ is defined to the ${\widehat{\mathbb{Q}}}_{\alpha }={\mathbb{Q}}_{\alpha }\left(\widehat{\theta }\right)$. Therefore, the parametric maximum likelihood estimators of the supersaturated PCI is

6 $${\widehat{C}}_{Np}\left(\eta ,\kappa ,\widehat{\theta }\right)=\frac{d-\eta \left|{\mathbb{Q}}_{{p}_{2}}(\widehat{\theta })-m\right|}{3\sqrt{{\left[\frac{{\mathbb{Q}}_{{p}_{3}}(\widehat{\theta })-{\mathbb{Q}}_{{p}_{1}}(\widehat{\theta })}{6}\right]}^{2}+\kappa {\left({\mathbb{Q}}_{{p}_{2}}(\widehat{\theta })-T\right)}^{2}}}$$

Note that ${C}_{Np}\left(\eta ,\kappa ,\theta \right)$ is a real-valued function of quantile, ${\mathbb{Q}}_{{p}_{1}},{\mathbb{Q}}_{{p}_{2}},$ and ${\mathbb{Q}}_{{p}_{3}}$ which are a continuous real-valued function of the parameter $\theta$. Since $\widehat{\theta }$ is a consistent MLE of $\theta$, ${\widehat{C}}_{Np}\left(\eta ,\kappa ,\widehat{\theta }\right)$ is a consistent MLE of ${C}_{Np}\left(\eta ,\kappa ,\theta \right)$ under some regularity conditions^40^*.*

### Median absolute deviation (MAD) based PCI

Suppose that the sample median (MD) is computed from a random sample $({x}_{1},{x}_{2},\dots \dots ,{x}_{n})$. Then MAD from the sample median is defined as^25,26,41^.

7 $$MAD=b*median\left\{\left|{x}_{i}-MD\right|\right\} .$$

The value of constant b in (7) is used to make it as a consistent estimator. In case of normal distribution, *MAD* is an unbiased estimator of $\sigma$ if $b=1.4826$. For any non-normal distribution, this value changes to $b={Q}_{0.75}^{-1}$*,* where ${Q}_{0.75}$ is the ${75}^{th}$ quantile of any underlying distribution. In case of normality, ${Q}_{0.75}^{-1}=1.4826$. Thus, the unbiased estimator of $\sigma$ is

8 $$\widehat{\sigma }=1.4826\left(MAD\right)$$

Using (8) the MAD based estimators for supersaturated and third generation PCIs can be defined as

9 $${\widehat{C}}_{MAD}\left(\eta ,k\right)=\frac{d-\eta \left|M-m\right|}{3\sqrt{{\widehat{\sigma }}^{2}+k{\left(M-T\right)}^{2}}}$$

10 $${\widehat{C}}_{pmMAD}=\frac{USL-LSL}{6\sqrt{{\widehat{\sigma }}^{2}+{\left(M-T\right)}^{2}}}$$

11 $${\widehat{C}}_{pmkMAD}=\frac{\mathrm{min}(USL-M,M-LSL)}{3\sqrt{{\widehat{\sigma }}^{2}+{\left(M-T\right)}^{2}}}$$

### Inter quantile range (IQR) based PCI

The population IQR for any continuous distribution is defined as

12 $$IQR={Q}_{3}-{Q}_{1}$$

where both upper and lower quantiles are found by solving the following integrals

13 $$\underset{-\infty }{\overset{{Q}_{3}}{\int }}f\left(x\right)dx=0.75 .$$

14 $$\underset{-\infty }{\overset{{Q}_{1}}{\int }}f\left(x\right)dx=0.25 .$$

Using (12) the IQR based estimators for supersaturated and third generation PCIs can be defined as

15 $${\widehat{C}}_{pmIQR}=\frac{USL-LSL}{2(IQR+\left|M-T\right|)}$$

16 $${\widehat{C}}_{pmkIQR}=\frac{\mathrm{min}(USL-M,M-LSL)}{2(IQR+\left|M-T\right|)}$$

### Gini’s mean difference (GMD) based PCI

The Gini’s Mean Difference for a set of $n$ ordered observations, $\left\{{x}_{1},{x}_{2},\cdots ,{x}_{n}\right\}$ of a random variable $X$ which arranged in ascending order of magnitude, is defined as

17 $${G}_{n}=\frac{2}{n\left(n-1\right)}\sum_{j=1}^{n}\sum_{i=1}^{n}\left|{x}_{i}-{x}_{j}\right|.$$

18 $${G}_{n}=\frac{2}{n\left(n-1\right)}\sum_{i=1}^{n}\left[\left({x}_{i}-{x}_{1}\right)+\left({x}_{i}-{x}_{2}\right)+\cdots +\left({x}_{i}-{x}_{i-1}\right)\right]$$

19 $${G}_{n}=\frac{2}{n\left(n-1\right)}\sum_{i=1}^{n}\left(2i-n-1\right){x}_{(i)} .$$

If the random variable *x* follows normal distribution with mean $\mu$ and variance ${\sigma }^{2}$, then^42^, suggests as a possible unbiased estimator of standard deviation $(\sigma )$ is

20 $${\sigma }^{*}=c\sum_{i=1}^{n}\left(2i-n-1\right){x}_{(i)}/n\left(n-1\right) .$$

where $c=\sqrt{\pi }=1.77245$ and latter on^43^ proved that

21 $${\sigma }^{*}=0.8862* {G}_{n} .$$

is an unbiased measure of variability. Using (21) GMD based estimators for supersaturated and third generation PCIs can be defined as

22 $${\widehat{C}}_{pmGMD}=\frac{USL-LSL}{3\sqrt{{{\sigma }^{*}}^{2}+{(M-T)}^{2}}}$$

23 $${\widehat{C}}_{pmkGMD}=min\left[\frac{USL-M}{3\sqrt{{{\sigma }^{*}}^{2}+{(M-T)}^{2}}},\frac{M-LSL}{3\sqrt{{{\sigma }^{*}}^{2}+{(M-T)}^{2}}}\right]$$

### Case studies for non-normal distribution

One of the most suitable distribution that fits the quality parameters is Weibull distribution. The two parameter Weibull distribution, with $\gamma$ and $\beta$ as shape and scale parameters, is given as

24 $$f\left(z,\gamma ,\beta \right)=\frac{\gamma }{\beta }{\left(\frac{z}{\beta }\right)}^{\gamma -1}{e}^{-{\left(\frac{z}{\beta }\right)}^{\gamma }}$$

The cumulative distribution, quantile function for (24) respectively are defined as

25 $$F\left(z,\gamma ,\beta \right)=1-exp\left[{\left(-\frac{z}{\beta }\right)}^{\gamma }\right]$$

26 $${\mathbb{Q}}_{\alpha }=\beta {\left[-\mathit{ln}(1-\alpha )\right]}^{\frac{1}{\gamma }}$$

The maximum likelihood estimator of $\gamma$ and $\beta$ are defined as

27 $$\widehat{\gamma }={\left[\left(\frac{\sum_{i=1}^{n}{{z}_{i}}^{\gamma }\left(\mathit{ln}{z}_{i}\right)}{\sum_{i=1}^{n}{{z}_{i}}^{\gamma }}\right)-\frac{{\sum }_{i=1}^{n}\mathit{ln}{z}_{i}}{n}\right]}^{-1}$$

28 $$\widehat{\beta }={\left(\frac{\sum_{i=1}^{n}{{z}_{i}}^{\gamma }}{n}\right)}^{\frac{1}{\gamma }}.$$

### The IQR of Weibull process

The IQR for Weibull process defined in (24) is defined as

29 $${IQR}_{Wei}={\beta }^{\frac{1}{\gamma }}\left\{\mathit{ln}{\left(0.25\right)}^{\frac{1}{\gamma }}- ln{(0.75)}^{\frac{1}{\gamma }}\right\}$$

### The Gini’s mean difference of Weibull process

By following the procedure of^44^, the unbiased estimator of GMD for Weibull distribution is,

30 $$E\left({G}_{n}\right)=\left(2-{2}^{1-\frac{1}{\gamma }}\right)\frac{\Gamma \left(1+\frac{1}{\gamma }\right)}{1/\beta }={\sigma }_{gw}$$

**Figure 1:**
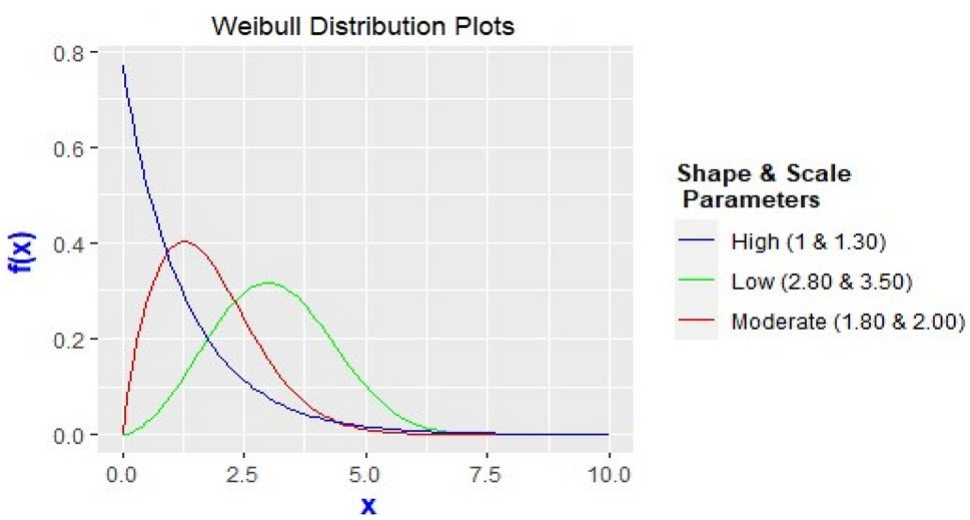
PDF plots of Weibull distributions with different asymmetry levels along with shape and scale parameters.

To evaluate the performance of robust third generation PCIs at different skewness behaviour of Weibull distribution, shape and scale parameters are selected so that the skewness level may be categorized as low, moderate and high as shown in Fig. 1.

### Methods of bootstrap confidence interval

The bootstrap technique originated from^45^. Morove Efron^45^ and Hall et al.^46^ provide theoretical details about the bootstrap technique. This technique can be used to construct confidence intervals for parameters when the usual interval estimation approach is not feasible. BCIs are commonly applied in constructing the confidence intervals for various PCIs. Suppose that ${\varsigma }_{1}, {\varsigma }_{2}, ..., {\varsigma }_{n}$ constitute a random sample with n observations taken from a distribution of interest, say $\upphi$, i.e. ${\varsigma }_{1}, {\varsigma }_{2}, \dots , {\varsigma }_{n}\sim\upphi$. Let $\widehat{\uptheta }$ represent an estimator of an arbitrary PCIs say ${C}_{pm or }{C}_{pmk}$.

Then the bootstrap technique is implemented as follows:

i. A bootstrap sample with n observations (with replacement) is taken from the original sample by using $\frac{1}{n}$ as the mass at each point, where this bootstrap sample is denoted as ${\varsigma }_{1}^{*},{\varsigma }_{2}^{*},\dots ,{\varsigma }_{n}^{*}$.
ii. From the kth bootstrap sample, for $1\le k\le n$, the kth bootstrap estimator of θ (an arbitrary PCI) can be denoted as ${\widehat{\uptheta }}^{*}=\widehat{\uptheta }\left({\varsigma }_{1}^{*},{\varsigma }_{2}^{*},\dots \dots ,{\varsigma }_{n}^{*}\right).$
iii. If the number of resamples in the bootstrap technique is B, then a total of B estimates of ${\widehat{\uptheta }}^{*}$ can be obtained. Arranging the whole collection from the smallest to the largest value constitutes an empirical bootstrap distribution of $\widehat{\uptheta }$^13^. B = $1000$ bootstrap resamples is considered in this article. The confidence intervals of $\widehat{\uptheta }$ can be constructed using any of the following three bootstrap techniques.

#### Method 1: Standard bootstrap (SB) confidence interval

The sample average and sample standard deviation are computed as follows using the 1000 bootstrap estimates of ${\widehat{\uptheta }}^{*}$:

31 $${\overline{\uptheta } }^{*}={\left(1000\right)}^{-1}\sum_{i=1}^{1000}{\widehat{\uptheta }}^{*}$$

32 $${s}_{{\widehat{\uptheta }}^{*}}=\sqrt{\frac{1}{999}\sum_{i=1}^{1000}{\left({\widehat{\uptheta }}^{*}\left(i\right)-{\overline{\uptheta } }^{*}\right)}^{2}}$$

Consequently, the 1$00\left(1-\alpha \right)\mathrm{\%}$ SB confidence interval is obtained as

33 $${\mathrm{CI}}_{\mathrm{SB}}={\overline{\uptheta } }^{*}\pm {z}_{\left(1-\frac{\alpha }{2}\right)} {s}_{{\widehat{\uptheta }}^{*}},$$

where ${z}_{\left(1-\frac{\alpha }{2}\right)}$ is the ${\left(1-\frac{1}{\alpha }\right)}{\mathrm{th}}$ quantile of the standard normal variable.

#### Method 2: Percentile bootstrap (PB) confidence interval

Since there is a total of $B$ resamples of ${\widehat{\uptheta }}^{*}$, these resamples will produce $B$ estimates of ${\widehat{\uptheta }}^{*}$. An arrangement of these estimates from the smallest value to the largest value will form an empirical distribution of ${\widehat{\uptheta }}^{*}$. From the ordered empirical distribution of ${\widehat{\uptheta }}^{*}$, choose the $100\left(\frac{\alpha }{2}\right)$ and $100\left(1-\frac{\alpha }{2}\right)$ percentiles as the end points of the interval, which results in the $100\left(1-\alpha \right)\mathrm{\%}$ PB confidence interval for ${\widehat{\uptheta }}^{*}$ given as

34 $${\mathrm{CI}}_{\mathrm{PB}}=\left({{\widehat{\uptheta }}^{*}}_{1000\left(\frac{\alpha }{2}\right)},{{\widehat{\uptheta }}^{*}}_{1000\left(1-\frac{\alpha }{2}\right)}\right)$$

For example, the $95\mathrm{\%}$ confidence interval with 1000 bootstrap estimates is

35 $${\mathrm{CI}}_{\mathrm{PB}}=\left({{\widehat{\uptheta }}^{*}}_{\left(25\right)},{{\widehat{\uptheta }}^{*}}_{\left(975\right)}\right)$$

where ${{\widehat{\uptheta }}^{*}}_{\left(25\right)}$ and ${{\widehat{\uptheta }}^{*}}_{\left(975\right)}$ represent the 25th and 975th ordered collection of the bootstrap estimates of ${\widehat{\uptheta }}^{*}$.

#### Method 3: Bias-corrected percentile bootstrap (BCPB) confidence interval

This technique was established to address the potential bias that could occur as the bootstrap distribution is based on a sample from the complete bootstrap distribution, which may be shifted higher or lower than would be expected. The following steps explain the implementation of this technique:

i. By means of the (ordered) distribution of ${\widehat{\uptheta }}^{*}$, calculate
  36 $${l}_{0}=\mathrm{Pr}\left({\widehat{\uptheta }}^{*}\le \widehat{\uptheta }\right)$$
ii. By letting ${\rho }^{-1}$ as the inverse distribution function of the standard normal variable, calculate
  37 $${q}_{0}={\rho }^{-1}({l}_{0})$$
iii. The lower percentile and upper percentile of the ordered distribution of ${\widehat{\uptheta }}^{*}$ are
  38 $${P}_{L}=\rho \left(2{q}_{0}+{z}_{\left(\frac{\alpha }{2}\right)}\right)$$
  and
  39 $${P}_{U}=\rho \left(2{q}_{0}+{z}_{\left(1-\frac{\alpha }{2}\right)}\right)$$
  respectively, where ρ, ${z}_{\left(\frac{\alpha }{2}\right)}$ and ${z}_{\left(1-\frac{\alpha }{2}\right)}$ are the distribution function, ${\left(\frac{\alpha }{2}\right)}{\mathrm{th}}$ quantile and ${\left(1-\frac{\alpha }{2}\right)}{\mathrm{th}}$ quantile, respectively, of the standard normal distribution. Consequently, the $100\left(1-\alpha \right)\mathrm{\%}$ BCPB confidence interval is constructed as
  40 $${\mathrm{CI}}_{\mathrm{BCPB}}=\left({{\widehat{\uptheta }}^{*}}_{1000\left({P}_{L}\right)},{{\widehat{\uptheta }}^{*}}_{1000\left({P}_{U}\right)}\right)$$

The average width (AW) is considered to compare the three different types of BCIs. The AW of the BCI is computed using a total of $M$ trials. Next, the estimated AW is computed as

41 $$\mathrm{AW}=\frac{\sum_{i=1}^{M}({U}_{{p}_{i}}-{L}_{{w}_{i}})}{M}$$

where ${L}_{{w}_{i}}$ and ${U}_{{w}_{i}}$ are the estimated lower confidence limit and upper confidence limit of the 100 $(1-\alpha )\mathrm{\%}$ confidence interval for any of the three types of BCIs based on the ith replicate.

## Results and discussion

The point and interval estimation of modified PCIs based on Quantile (PC), MAD, IQR and GMD for different asymmetric behavior of Weibull distribution is given in Tables 1, 2, 3, 4.

**Table 1:** The statistical indicators of index ${C}_{pm}$ and ${C}_{pmk}$ for different asymmetric level of Weibull process based on PC-method.

	$n$	${C}_{pm}$			${C}_{pmk}$		
		Low	Moderate	High	Low	Moderate	High
Mean (SD)	25	1.5237 (0.2053)	1.51261 (0.2657)	1.3263 (0.3990)	1.4424 (0.2110)	1.4196 (0.2546)	1.1932 (0.3444)
	50	1.4957 (0.1375)	1.4721 (0.1806)	1.2574 (0.2674)	1.4419 (0.1418)	1.3988 (0.1750)	1.1368 (0.2351)
	75	1.4862 (0.1114)	1.4611 (0.1474)	1.2330 (0.2126)	1.4434 (0.1153)	1.3949 (0.1439)	1.1160 (0.1877)
	100	1.4829 (0.0956)	1.4552 (0.1226)	1.2179 (0.1801)	1.4452 (0.0989)	1.3927 (0.1246)	1.1033 (0.1599)
MSE (bias)	25	0.0375 (0.1601)	0.0334 (0.1790)	0.0000 (− 0.0028)	0.0126 (0.0929)	0.0080 (0.0879)	0.0187 (− 0.1052)
	50	0.0275 (0.1369)	0.0202 (0.1393)	0.0053 (− 0.0558)	0.0125 (0.0925)	0.0047 (0.0674)	0.0373 (− 0.1486)
	75	0.0244 (0.1291)	0.0172 (0.1286)	0.0094 (− 0.0764)	0.0129 (0.0937)	0.0042 (0.0363)	0.0458 (− 0.1646)
	100	0.0234 (0.1264)	0.0157 (0.1228)	0.0126 (− 0.0862)	0.0133 (0.0952)	0.0039 (0.0615)	0.0514 (− 0.1744)

**Table 2:** The statistical indicators of index ${C}_{pm}$ and ${C}_{pmk}$ using selected asymmetric level of Weibull process based on MAD-method.

Indicator	$n$	${C}_{pm}$			${C}_{pmk}$		
		Low	Moderate	High	Low	Moderate	High
Mean (SD)	25	1.4353 (0.4066)	1.4930 (0.4093)	1.8529 (0.4674)	1.3748 (0.4003)	1.4184 (0.3986)	1.7126 (0.4384)
	50	1.3562 (0.2616)	1.4037 (0.2637)	1.7759 (0.3218)	1.3147 (0.2587)	1.3451 (0.2562)	1.6412 (0.2952)
	75	1.3329 (0.2091)	1.3739 (0.2103)	1.7647 (0.2629)	1.2988 (0.2068)	1.3201 (0.2040)	1.6301 (0.2396)
	100	1.3189 (0.1768)	1.3621 (0.1768)	1.7491 (0.2302)	1.2893 (0.2068)	1.3105 (0.1710)	1.6159 (0.2083)
MSE (bias)	25	0.0111 (0.0870)	0.0266 (0.1598)	0.2735 (0.4022)	0.0020 (0.0370)	0.0078 (0.0867)	0.1465 (0.2943)
	50	0.0008 (0.0217)	0.0054 (0.0722)	0.1988 (0.3430)	0.0003 (− 0.0126)	0.0002 (0.0148)	0.0969 (0.2394)
	75	0.0000 (0.0024)	0.0020 (0.0431)	0.1889 (0.3344)	0.0010 (− 0.0258)	0.0001 (− 0.0097)	0.0901 (0.2309)
	100	0.0000 (− 0.0091)	0.0010 (0.0315)	0.1756 (0.3224)	0.0017 (− 0.0336)	0.0004 (− 0.0191)	0.0817 (0.2199)

**Table 3:** The statistical indicators of index ${C}_{pm}$ and ${C}_{pmk}$ using selected asymmetric level of Weibull process based on IQR-method.

Indicator	$n$	${C}_{pm}$			${C}_{pmk}$		
		Low	Moderate	High	Low	Moderate	High
Mean (SD)	25	2.9605 (0.8281)	2.9599 (0.8038)	3.2494 (0.8607)	2.8406 (0.8349)	2.8171 (0.8082)	3.0078 (0.8275)
	50	2.8289 (0.5401)	2.8060 (0.5248)	3.0483 (0.5607)	2.7457 (0.5495)	2.6921 (0.5353)	2.8206 (0.5351)
	75	2.7869 (0.4344)	2.7499 (0.4238)	2.9739 (0.4449)	2.7176 (0.4438)	2.6451 (0.4351)	2.7504 (0.4229)
	100	2.7843 (0.3787)	2.7281 (0.3561)	2.9410 (0.3812)	2.7236 (0.3884)	2.6285 (0.3675)	2.7179 (0.3606)
MSE (bias)	25	2.6586 (1.3475)	2.6566 (1.5979)	3.6840 (1.4764)	2.2820 (1.2485)	2.2115 (1.4580)	2.8150 (1.2906)
	50	2.2466 (1.2387)	2.1786 (1.4471)	2.9527 (1.3218)	2.0043 (1.1700)	1.8553 (1.3354)	2.2220 (1.1466)
	75	2.1224 (1.2040)	2.0160 (1.3920)	2.7025 (1.2646)	1.9421 (1.1517)	1.7295 (1.2893)	2.0175 (1.0926)
	100	2.1149 (1.2019)	1.9546 (1.3707)	2.5954 (1.2392)	1.9254 (1.1468)	1.6862 (1.2731)	1.9263 (1.0676)

**Table 4:** The statistical indicators of index ${C}_{pm}$ and ${C}_{pmk}$ using selected asymmetric level of Weibull process based on GMD-method.

Indicator	$n$	${C}_{pm}$			${C}_{pmk}$		
		Low	Moderate	High	Low	Moderate	High
Mean (SD)	25	1.8424 (0.2821)	1.5221 (0.2492)	2.1500 (0.5351)	1.7667 (0.2945)	1.4483 (0.2597)	2.0039 (0.5637)
	50	1.9145 (0.2048)	1.5651 (0.1802)	2.1877 (0.3918)	1.8567 (0.2135)	1.5006 (0.1920)	2.0334 (0.4186)
	75	1.9388 (0.1664)	1.5838 (0.1460)	2.1973 (0.3316)	1.8896 (0.1748)	1.5226 (0.1580)	2.0380 (0.3544)
	100	1.9539 (0.1463)	1.5901 (0.1266)	2.1966 (0.2833)	1.9106 (0.1525)	1.5307 (0.1381)	2.0348 (0.3023)
MSE (bias)	25	0.2626 (0.4235)	0.0369 (0.1884)	0.6724 (0.6307)	0.1907 (0.3609)	0.0140 (0.1160)	0.4542 (0.5184)
	50	0.3416 (0.4830)	0.0553 (0.2305)	0.7358 (0.6598)	0.2774 (0.4353)	0.0291 (0.1673)	0.4948 (0.5411)
	75	0.3707 (0.5032)	0.0644 (0.2488)	0.7522 (0.6671)	0.3131 (0.4625)	0.0371 (0.1883)	0.5013 (0.5446)
	100	0.3893 (0.5156)	0.0677 (0.2550)	0.7509 (0.6666)	0.3371 (0.4798)	0.0403 (0.1968)	0.4967 (0.5421

Following^47^ target values equal to 1.33 corresponding to existing processes were considered for the point estimation of indices ${C}_{pm}$, and ${C}_{pmk}$. The performance of each modified PCI under different asymmetric behavior is evaluated by using 10,000 simulated samples of size 25, 50,75 and 100. The R-Statistical language was used to complete simulation study. Bias and Mean Square Error (MSE) criteria has been used for the comparison purpose. The simulations have been performed on the following steps

1. Collect 10,000 samples of size 25 from Weibull process with parameters $\left[\left(Shape, Scale\right)= \left(2.8, 3.5\right), \left(\mathrm{1.80,2.00}\right), (\mathrm{1.00,1.30})\right]$.
2. Compute ${\widehat{C}}_{pm}$ & ${\widehat{C}}_{pmk}$ based on the measures of MAD (Median Absolute Deviation), IQR (Interquartile Range) and GMD (Ginni' s Mean Differnce)$.$
3. Calculate average and standard deviation of the computed PCIs.
4. Repeat the entire process for sample size of 50, 75 and 100.

### Results for PC based PCIs

Simulation results of quantile approach as suggested by^23^ are presented in Table 1 for Weibull distribution. These tables depict the simulated mean, MSE, standard deviation and bias in parenthesis, bias and mean square error (MSE) corresponding to the target value equal to 1.33 for both indices for low, moderate and high asymmetric behavior of Weibull distribution.

In the case of index ${C}_{pm},$ the PC-method gives good results under low and moderate asymmetric behavior, however, underestimates the target value in case of high asymmetry. As the sample size increases, the estimated values come close to the target values and ultimately produce less bias and mean square error. For the index ${C}_{pmk}$, the PC-method is more accurate as compared to other three indices and gives lowest bias and MSE under low and moderate asymmetric conditions for the sample $(n=100)$. For the three asymmetric levels of the Weibull distribution, following conclusions can be drawn; the PC-method gives a lower bias and MSE for indices ${C}_{pmk}$ under lower and moderate asymmetric behaviors when the target value is $1.33$.

### Results of MAD base PCIs

Table 2 summarize the results of MAD- based estimators of both PCIs i.e., ${C}_{pm}$ and ${C}_{pmk}$. Unlike the PC-method, MAD-based estimators of two indices showed a different pattern for Weibull process. Summing up the overall results, it can be concluded that performance of MAD based estimators is consistently better than that of PC-based estimator from low to high asymmetry.

For index ${C}_{pm}$, except for high asymmetry, the MAD-based estimator is closer to the target value and less biased for large samples. The MAD-based estimator of index ${C}_{pmk}$ showed good performance for small sample sizes only. It showed accurate results under low and moderate asymmetric condition whereas for the new process it deals better with high asymmetry. In both cases, it slightly underestimates the target values for a large sample.

### Results for IQR based PCIs

The simulation results of IQR based PCIs ${C}_{pm}$, and ${C}_{pmk}$ for Weibull distribution under low, moderate and high asymmetric levels are reported in Table 3. The simulation results of both indices using IQR-method for all asymmetric levels of three distributions show the overestimation using all sample sizes. So, these estimators do not consider as good estimators. In all cases, large bias and MSE for all sample sizes is observed. The situation tends to worse estimation for all indices as asymmetry level turns from low to high level. Moreover, the findings of the simulation results indicate that IQR-method could not be a useful and attractive method for practical point of view due to large bias and MSE.

### Results for GMD based PCIs

In this section, the performance of both PCIs, ${C}_{pm}$ and ${C}_{pmk}$ based on GMD-method has been assessed and compared under low, moderate and high asymmetric condition of Weibull distribution. The results are presented in Table 4. The results indicate that GMD-based PCIs perform better under the moderate asymmetric condition for the index ${C}_{pm}$ for large samples. The bias and MSE reduce as sample size increases. In case of index ${C}_{pmk}$, the GMD-based estimator slightly overestimates the target value of 1.67 for small samples under low asymmetry, but bias increases as sample size increases. For moderate asymmetry this method underestimates, and for high asymmetry, it again overestimates the target value of new processes. Based on the above observations, GMD-based estimators of indices ${C}_{pm}$ and ${C}_{pmk}$ have the following results

1. The GMD-method performed well for new processes under moderate conditions for large samples sizes for index ${C}_{pm}$.
2. In case of index ${C}_{pmk}$, this method is good for small samples under low asymmetric conditions.
3. As compared to other methods, in case of GMD-method, the mean estimated values increases as sample size increases. However, for the efficient process in which there is a very low amount of product is outside the specification limits, GMD is recommended under high asymmetry.

### Bootstrap confidence intervals for ${{\varvec{C}}}_{{\varvec{p}}{\varvec{m}}}$ and ${{\varvec{c}}}_{{\varvec{p}}{\varvec{m}}{\varvec{k}}}$

In this section, four bootstrap confidence intervals, namely standard, percentile, bias-corrected percentile and percentile-t bootstrap confidence intervals are discussed for indices ${C}_{pm}$ and ${C}_{pmk}$ using PC, MAD and GMD method. For the simulation, Weibull process are used under low, moderate and high asymmetric conditions for sample sizes n = 25, 50, 75 $\text{and 100}$. The results are presented in Tables 5, 6, 7, 8, 9, 10 which indicate true index value, 95% confidence limits, and coverage probability of each index under low, moderate and high asymmetric conditions for all sample sizes. These results are based on 1000 replications and different values of USL and LSL for the three types of processes which are given in Table 2 above.

**Table 5:** The 95% bootstrap confidence intervals with coverage probabilities for Weibull distribution using PC methods for index ${C}_{pm}$.

$n$	${C}_{pm}$	SB	PB	BCPB	PTB
Low asymmetry					
25	1.4698	(1.1651–1.9902)	(1.2153–2.0443)	(1.1738–1.9472)	(1.2131–2.0440)
		0.9300	0.8870	0.9230	0.8880
50	1.4698	(1.4106–2.0276)	(1.4485–2.0576)	(1.4189–1.9837)	(1.4482–2.0576)
		0.9500	0.9210	0.9490	0.9220
75	1.4698	(1.2606–1.6766)	(1.2697–1.6879)	(1.2604–1.6779)	(1.2690–1.6878)
		0.9550	0.9350	0.9470	0.9380
100	1.4698	(1.3014–1.6658)	(1.3164–1.6703)	(1.3088–1.6658)	(1.3113–1.6703)
		0.9550	0.9450	0.9510	0.9450
Moderate asymmetry					
25	1.4363	(1.0654–2.1746)	(1.1521–2.2337)	(1.0559–2.0786)	(1.1427–2.2336)
		0.9210	0.8770	0.9240	0.8810
50	1.4363	(1.3643–2.1552)	(1.4059–2.1740)	(1.3731–2.0997)	(1.4059–2.1737)
		0.9470	0.9180	0.9450	0.9180
75	1.4363	(1.1620–1.7091)	(1.1747–1.7261)	(1.1548–1.7024)	(1.1741–1.7261)
		0.9510	0.9360	0.9490	0.9370
100	1.4363	(1.2166–1.6956)	(1.2312–1.7033)	(1.2251–1.6938)	(1.2298–1.7032)
		0.950	0.9450	0.9500	0.9470
High asymmetry					
25	1.1859	(0.6305–2.4033)	(0.8222–2.5510)	(0.6989–2.2146)	(0.8096–2.5507)
		0.9260	0.8650	0.9170	0.8720
50	1.1859	(1.0577–2.3025)	(1.1503–2.3454)	(1.0957–2.2058)	(1.1479–2.3449)
		0.9480	0.9140	0.9430	0.9180
75	1.1859	(0.8015–1.5847)	(0.8376–1.6159)	(0.8135–1.5858)	(0.8366–1.6159)
		0.9560	0.9330	0.9500	0.9360
100	1.1859	(0.8765–1.5654)	(0.9122–1.5944)	(0.9024–1.5711)	(0.9101–1.5943)
		0.9520	0.9400	0.9480	0.9450

**Table 6:** The 95% bootstrap confidence intervals with coverage probabilities for weibull distribution using PC methods for index ${C}_{pmk}$.

$n$	${C}_{pmk}$	SB	PB	BCPB	PTB
Low asymmetry					
25	1.4554	(0.9733–1.8714)	(1.0207–1.9094)	(1.0054–1.8893)	(1.0173–1.9090)
		0.9660	0.9440	0.9390	0.9450
50	1.4554	(1.3495–1.9846)	(1.3875–1.9991)	(1.4141–2.0399)	(1.3861–1.9990)
		0.9670	0.9640	0.9580	0.9650
75	1.4554	(1.2105–1.6442)	(1.2282–1.6597)	(1.2722–1.7527)	(1.2256–1.6596)
		0.9720	0.9720	0.9700	0.9720
100	1.4554	(1.2560–1.6359)	(1.2710–1.6420)	(1.2949–1.6728)	(1.2636–1.6420)
		0.9610	0.9590	0.9560	0.9590
Moderate asymmetry					
25	1.3793	(0.9006–1.9330)	(0.9649–1.9944)	(0.9065–1.9005)	(0.9642–1.9944)
		0.9400	0.9180	0.9250	0.9180
50	1.3793	(1.2952–2.0763)	(1.3380–2.0837)	(1.3065–2.0313)	(1.3354–2.0837)
		0.9550	0.9450	0.9390	0.9470
75	1.3793	(1.1087–1.6442)	(1.1191–1.6589)	(1.1196–1.6589)	(1.1148–1.6589
		0.9640	0.9550	0.9580	0.9550
100	1.3793	(1.1533–1.6284)	(1.1665–1.6435)	(1.1662–1.6431)	(1.1656–1.6435)
		0.9580	0.9500	0.9440	0.9510
High asymmetry					
25	1.0751	(0.5711–1.9452)	(0.7001–2.0426)	(0.6175–1.8186)	(0.6993–2.0426)
		0.9240	0.8810	0.9170	0.8820
50	1.0751	(0.9739–2.0683)	(1.0468–2.1030)	(0.9806–1.9341)	(1.0401–2.1027)
		0.9460	0.9140	0.9380	0.9180
75	1.0751	(0.7394–1.4334)	(0.7655–1.4683)	(0.7412–1.4328)	(0.7645–1.4682)
		0.9560	0.9360	0.9530	0.9390
100	1.0751	(0.7961–1.4105)	(0.8192–1.4461)	(0.7943–1.4058)	(0.8194–1.4461)
		0.9490	0.9380	0.9490	0.9380

**Table 7:** The 95% bootstrap confidence intervals with coverage probabilities Weibull distribution using MAD methods for index ${C}_{pm}$.

$n$	${C}_{pm}$	SB	PB	BCPB	PTB
Low asymmetry					
25	1.6850	(0.7549–2.7134)	(1.0117–2.9333)	(1.0776–3.2092)	(1.0024–2.9330)
		0.9950	0.9970	0.6410	0.9970
50	1.3511	(0.9347–1.9961)	(1.0374–2.1091)	(1.0981–2.2579)	(1.0359–2.1089)
		0.9990	0.9990	0.7290	0.9990
75	1.3106	(0.8629–1.5873)	(0.9056–1.6296)	(1.3334–1.8542)	(0.9056–1.6292)
		0.9990	0.9990	0.7010	0.9990
100	1.3890	(1.0642–1.8442)	(1.1250–1.9052)	(1.3114–2.2145)	(1.1241–1.9051)
		0.9990	0.9990	0.7170	0.9990
Moderate asymmetry					
25	1.3241	(0.8180–2.7150)	(1.0610–2.9917)	(1.2165–3.5732)	(1.0459–2.9913)
		0.9990	0.9890	0.6780	0.9890
50	1.5686	(0.9721–2.0213)	(1.0816–2.1281)	(1.0837–2.1358)	(1.0751–2.1281)
		0.9970	0.9990	0.6580	0.9990
75	1.2530	(0.8630–1.6250)	(0.9132–1.6991)	(1.2205–1.9495)	(0.9124–1.6991)
		0.9980	0.9890	0.7050	0.9900
100	1.4080	(1.1123–1.8855)	(1.1807–1.9295)	(1.3373–2.2095)	(1.1802–1.9292)
		0.9990	0.9990	0.7040	0.9990
High asymmetry					
25	1.6086	(1.1658–3.0107)	(1.3042–3.1582)	(1.5482–3.9350)	(1.3018–3.1572)
		0.9970	0.9860	0.6620	0.9860
50	2.3319	(1.2383–2.4590)	(1.2835–2.4720)	(1.2174–2.3975)	(1.2832–2.4717)
		0.7080	0.8010	0.5240	0.8010
75	1.6320	(1.0152–2.1191)	(1.0803–2.1536)	(1.3098–2.5419)	(1.0782–2.1535)
		0.9960	0.9960	0.6820	0.9960
100	1.7448	(1.4418–2.3105)	(1.4757–2.3331)	(1.6590–2.5127)	(1.4718–2.3332)
		0.9990	0.9990	0.6930	0.9990

**Table 8:** The 95% bootstrap confidence intervals with coverage probabilities Weibull distribution using MAD methods for index ${C}_{pmk}$.

$n$	${C}_{pmk}$	SB	PB	BCPB	PTB
Low asymmetry					
25	1.2356	(0.7060–2.6356)	(0.9548–2.8347)	(1.0699–3.4150)	(0.9528–2.8341)
		0.9990	0.9880	0.7170	0.9890
50	1.3382	(0.9047–1.9467)	(1.0083–2.0613)	(1.1229–2.3601)	(0.9994–2.0610)
		0.9990	0.9990	0.7400	0.9990
75	1.2981	(0.8120–1.5375)	(0.8517–1.5681)	(1.2662–1.7983)	(0.8514–1.5679)
		0.9990	0.9990	0.7200	0.9990
100	1.3758	(1.0311–1.8081)	(1.0801–1.8654)	(1.3041–2.1524)	(1.0782–1.8653)
		0.9950	0.9990	0.7290	0.9990
Moderate asymmetry					
25	1.2764	(0.7482–2.6184)	(0.9888–2.9091)	(1.1589–3.4544)	(0.9868–2.9088)
		0.9990	0.9910	0.7000	0.9910
50	1.5121	(0.9354–1.9541)	(1.0337–2.0592)	(1.0449–2.1236)	(1.0269–2.0588)
		0.9890	0.9960	0.6710	0.9960
75	1.2079	(0.8283–1.5918)	(0.8723–1.6497)	(1.2253–1.8861)	(0.8721–1.6496)
		0.9990	0.9930	0.7150	0.9930
100	1.3573	(1.0564–1.8088)	(1.1181–1.8412)	(1.2883–2.1193)	(1.1168–1.8412)
		0.9990	0.9990	0.7180	0.9990
High asymmetry					
25	1.4849	(1.0335–2.8191)	(1.2165–3.0159)	(1.4586–3.8360)	(1.2129–3.0157)
		0.9990	0.9880	0.6650	0.9890
50	2.1526	(1.1548–2.2882)	(1.2131–2.3258)	(1.3118–2.2236)	(1.2103–2.3257)
		0.6590	0.7940	0.5320	0.7940
75	1.5065	(0.9831–2.0209)	(1.0445–2.0683)	(1.2746–2.4786)	(1.0434–2.0681)
		0.9990	0.9990	0.6680	0.9990
100	1.6106	(1.3212–2.1212)	(1.3559–2.1524)	(1.5212–2.3450)	(1.3526–2.1524)
		0.9990	0.9990	0.6820	0.9990

**Table 9:** The 95% bootstrap confidence intervals with coverage probabilities for Weibull distribution using GMD methods for index ${C}_{pm}$.

$n$	${C}_{pm}$	SB	PB	BCPB	PTB
Low asymmetry					
25	1.9943	(1.1839–2.1826)	(1.2355–2.2286)	(1.6804–2.5488)	(1.2350–2.2284)
		0.7800	0.7940	0.8630	0.7940
50	1.9943	(1.2926–1.9813)	(1.3112–1.9795)	(1.4245–2.0751)	(1.2999–1.9795)
		0.8620	0.8690	0.9110	0.8690
75	1.9943	(1.6411–2.2868)	(1.6496–2.3191)	(1.7458–2.4024)	(1.6493–2.3191)
		0.9040	0.9100	0.9290	0.9100
100	1.9943	(1.6762–2.2282)	(1.6811–2.2247)	(1.7463–2.3232)	(1.6799–2.2245)
		0.9110	0.9150	0.9180	0.9150
Moderate asymmetry					
25	1.6168	(0.9099–1.7562)	(0.9510–1.7821)	(1.2663–2.1426)	(0.9478–1.7820)
		0.8090	0.8190	0.9120	0.8190
50	1.6168	(1.0442–1.6258)	(1.0494–1.6232)	(1.1305–1.6979)	(1.0469–1.6230)
		0.8880	0.8900	0.9400	0.8900
75	1.6168	(1.3248–1.9010)	(1.3365–1.9099)	(1.3803–1.9487)	(1.3363–1.9097)
		0.9170	0.9230	0.9550	0.9230
100	1.6168	(1.3494–1.8315)	(1.3570–1.8418)	(1.3880–1.8808)	(1.3558–1.8418)
		0.9150	0.9220	0.9580	0.9220
High asymmetry					
25	2.2016	(0.8183–2.3964)	(0.9231–2.5203)	(1.4090–3.3803)	(0.9134–2.5201)
		0.8840	0.9030	0.9680	0.9030
50	2.2016	(1.3227–2.4681)	(1.3586–2.5004)	(1.4367–2.6364)	(1.3435–2.5001)
		0.9280	0.9390	0.9780	0.9390
75	2.2016	(1.6003–2.9287)	(1.6822–2.9891)	(1.6710–2.9833)	(1.6728–2.9890)
		0.9350	0.9440	0.9960	0.9450
100	2.2016	(1.6483–2.7022)	(1.7111–2.7622)	(1.7396–2.8331)	(1.7094–2.7622)
		0.9220	0.9330	0.9940	0.9350

**Table 10:** The 95% bootstrap confidence intervals with coverage probabilities for Weibull distribution using GMD methods for index ${C}_{pmk}$.

$n$	${C}_{pmk}$	SB	PB	BCPB	PTB
Low asymmetry					
25	1.9752	(1.0094–2.1142)	(1.0459–2.1601)	(1.2499–2.4180)	(1.0439–2.1600)
		0.7330	0.7600	0.8810	0.7600
50	1.9752	(1.2475–1.9406)	(1.2551–1.9416)	(1.4418–2.0930)	(1.2538–1.9416)
		0.8370	0.8510	0.9190	0.8510
75	1.9752	(1.5711–2.2543)	(1.5740–2.2701)	(1.7515–2.4090)	(1.5708–2.2700)
		0.8830	0.8910	0.9430	0.8910
100	1.9752	(1.6213–2.1952)	(1.6181–2.1903)	(1.7194–2.3028)	(1.6173–2.1903)
		0.8830	0.8880	0.9370	0.8880
Moderate asymmetry					
25	1.5586	(0.7536–1.6695)	(0.8098–1.7033)	(0.9204–1.9741)	(0.8080–1.7031)
		0.8020	0.8180	0.9020	0.8180
50	1.5586	(0.9916–1.5839)	(0.9972–1.6045)	(1.0865–1.6691)	(0.9855–1.6045)
		0.8880	0.8950	0.9110	0.8950
75	1.5586	(1.2433–1.8697)	(1.2482–1.8664)	(1.3259–1.9342)	(1.2466–1.8663)
		0.9270	0.9280	0.9330	0.9280
100	1.5586	(1.2587–1.7949)	(1.2729–1.8054)	(1.2917–1.8377)	(1.2716–1.8054)
		0.9180	0.9250	0.9280	0.9250
High asymmetry					
25	2.0322	(0.6327–2.2261)	(0.7685–2.3532)	(0.8566–2.7143)	(0.7611–2.3523)
		0.8940	0.9160	0.8710	0.9160
50	2.0322	(1.1603–2.3715)	(1.2321–2.4308)	(1.3061–2.6090)	(1.2300–2.4308)
		0.9290	0.9440	0.9130	0.9450
75	2.0322	(1.3962–2.8201)	(1.4870–2.9124)	(1.5264–2.9711)	(1.4781–2.9122)
		0.9280	0.9440	0.9380	0.9460
100	2.0322	(1.4502–2.5718)	(1.5251–2.6254)	(1.5293–2.6289)	(1.5195–2.6253)
		0.9270	0.9390	0.9410	0.9390

Tables 5 to 6, present the 95% BCIs for the Weibull process using PC-method, while the coverage probability of each method is reported below each interval. Similarly, Tables 7 to 8 presents the 95% BCIs for Weibull process along with coverage probabilities using the MAD method. The results presented in all these tables indicate that the average width of all confidence intervals, which is the difference between lower and upper specification limit, reduces when the sample size increases in all cases under study. Moreover, the asymmetric levels effect the average width, where the average width increases as asymmetry increases.

### BCIs for Weibull distribution

From the results of Weibull distribution, followings conclusions have been drawn.

i. Among the PC-based estimators of both indices ${C}_{pm}$ and ${C}_{pmk}$, BCBP method explicated least average width, under low, moderate and high asymmetric behavior of Weibull process.
ii. Based on the average with, the four bootstrap methods are ranked as *BCPB* < *PB* < *PTB* < *SB*.
iii. The coverage probability is directly proportional to sample size and reached to the nominal level 0.95 for large sample size in the case of SB and ${\text{BCPB}}$ method. However, other two methods did not reach to a nominal level, particularly for small samples.
iv. In the case of the MAD method, both ${\text{BCPB}}$ and PB CIs showed less average width as compared to SB and PTB. Based on the average with, the four bootstrap methods are ranked as *BCPB* < *PB* < *PTB* < *SB*.
v. Among ${\text{BCPB}}$ and PB CIs, former showed lower coverage probability than later. Consequently, PB CI performed better for MAD-method.
vi. In both methods, when the transition is made from low to high asymmetric conditions the average width approximately increased by two times. It means under high asymmetry; the width of CI is larger as compared to low and moderate asymmetry.

In general, ${\text{BCPB}}$ CI is recommended for all asymmetric condition when PC-method is used. On the other hand, PB CI is recommended for MAD-method under low, moderate and high asymmetric behavior of Weibull process. The recommendation is made on the basis of low average width and high coverage probability among four BCIs.

### Application of proposed methodology using practical data

A data sets was analysed using GMD, MAD and PC based PCIs. The results are appended in the following section.

### Data: strength measures in GPA for single fibres data

In this section, a real-life example is presented to demonstrate the application of the MAD, PC and GMD- methods for the indices ${C}_{pm}$, and ${C}_{pmk}$. The data which represents the strength measures in GPA for single fibres and impregnated 1000-carbon fibre tows. Single fibres were tested under tension at a gauge length of 20 mm with sample size $n=69$ and are given in Table 11^48–50^.

**Table 11:** Data set of strength measure in GPA for single fibre (20-mm).

1.312	1.314	1.479	1.552	1.700	1.803	1.861	1.865	1.944	1.958
1.966	1.997	2.006	2.021	2.027	2.055	2.063	2.098	2.140	2.179
2.224	2.240	2.253	2.270	2.272	2.274	2.301	2.301	2.359	2.382
2.382	2.426	2.434	2.435	2.478	2.490	2.511	2.514	2.535	2.554
2.566	2.570	2.586	2.626	2.633	2.642	2.648	2.684	2.697	2.726
2.770	2.773	2.800	2.809	2.818	2.821	2.848	2.880	2.954	3.012
3.067	3.084	3.090	3.096	3.128	3.233	3.433	3.858	3.585	

To select the appropriate distribution, the different goodness of fit statistics^51^ were used and reported in Table 12 along with summary statistics of the data. Based on AIC and BIC values, it is confirmed that two- parameter Weibull distribution is suitable for this data as compared to other distributions. By fitting two- parameter Weibull distribution, the maximum likelihood estimator for shape and scale parameters are $\widehat{\upgamma }=$ 5.504809, $\widehat{\upbeta }=$ 2.650830, respectively.

**Table 12:** The summary statistics and goodness of fit statistics for fibre strength data.

Statistic	Value	Statistic	Value
$n$	69	Kurtosis	3.03
Minimum	1.31	KS-Stat. Log-Normal	0.072
Maximum	3.58	KS-Stat. Gamma	0.058
Mean	2.45	KS-Stat. Weibull	0.056
S.D.	0.49	AIC. Log-Normal	106.76
${Q}_{0.00135}$	1.31	AIC. Gamma	104.07
${Q}_{0.50}$	2.48	AIC. Weibull	103.19
${Q}_{0.9986}$	3.58	BIC. Log-Normal	111.24
$MAD$	0.33	BIC. Gamma	108.54
$GMD$	0.56	BIC. Weibull	107.66
Skewness	− 0.03		

To evaluate the adequacy of the data K-S goodness of fit test is used. The K-S distance value for this data is 0.056 with p-value 0.9816, which also in favor of Weibull distribution. The lower and upper specification limits used for the calculations of PCIs were (0.3989, 4.4960). The estimates of both indices using three methods and their corresponding bootstrap CIs are reported in Table 13. Likewise, simulation study, the performance of MAD and GMD method are more accurate than PC-method. Both indices ${C}_{pk}$, ${C}_{pmk}$ showed better performance and estimated value is close to existing process target values. Based on the average width of CIs, the four bootstrap methods are ranked as ${\text{BCPB}} \, \text{<} \, {\text{PB}} \, \text{<} \, {\text{PTB}} \, \text{<} \, {\text{SB}}$. Overall, MAD and GMD, based estimator showed the wider spread of CIs.

**Table 13:** The point estimates and width of four BCIs for fibre strength data.

	MAD	PC	GMD
${C}_{pm}$	1.3888	1.8019	1.3711
SB	0.8971 (0.9174–1.8496)	0.3867 (1.2137–1.6111)	0.6191 (1.0448–1.6625)
PB	0.8890 (1.0230–1.9170)	0.3846 (1.2278–1.6310)	0.6148 (1.0858–1.7093)
BCPB	0.7948 (0.9151–1.6544)	0.3701 (1.2187–1.6103)	0.5613 (1.2003–1.9668)
PTB	0.8912 (1.0224–1.9162)	0.3858 (1.2273–1.6310)	0.6168 (1.0855–1.7093)
${C}_{pmk}$	1.3681	1.7743	1.3506
SB	0.8807 (0.8872–1.8043)	0.3836 (1.1713–1.5678)	0.6134 (1.0110–1.6223)
PB	0.8732 (0.9882–1.8691)	0.3819 (1.1827–1.5861)	0.6096 (1.0465–1.6694)
BCPB	0.7989 (0.9093–1.6668)	0.3871 (1.1967–1.6039)	0.5731 (1.1829–1.9297)
PTB	0.8755 (0.9870–1.8683)	0.3832 (1.1825–1.5861)	0.6115 (1.0463–1.6693)

## Summary and conclusion

Statistical Process Control (SPC) is an attractive statistical tool and commonly used to monitor the processes in many industries now a days. Among SPC, PCIs have become an attractive and important tool to measure the quality of any product within specified limits. It seems difficult to choose the proper PCI that performs accurately in non-normal distribution while process variability and mean are being affected by non-normality. Moreover, any PCI which does not provide high target value (> 1.33) even then its importance cannot be neglected. So, the conditions under which PCI performs poorly it opens a new research horizon for the researchers.

The pragmatic attempt has been conducted to address the non-normality issues in PCIs using quantile (PC), MAD, IQR and GMD methods under asymmetric conditions of Weibull distribution. Moreover, the point and interval estimation of modified PCIs were assessed using simulation studies. The point estimation of quantile-based PCIs using PC-method has been observed an effective approach under low and moderate asymmetric conditions of Weibull process. PC-based estimator tends to be an under-estimation. However, this trend increases as sample size increases. Results not only indicate that PC-based estimator produces large bias but also explain under and overestimation of target values. Moreover, the PC-based estimator is influenced by high asymmetry and explains the worst estimation for all three distributions.

The simulation studies reveal that the results of MAD-method can be successfully used and has a great potential to deal with non-normality for Weibull process under low and moderate asymmetry. Overall, MAD-based estimators tend to produce very accurate results under low and moderate asymmetric conditions. In the case of high asymmetry, MAD-estimator of index ${C}_{pm}$ has shown good performance only for a sample of size less than 50.

The simulation studies for PCIs show that IQR-method gives overestimation problem for selected asymmetric levels of Weibull distribution. Moreover, a large bias and MSE has been observed for all sample sizes. The situation became worse when asymmetry level turned from low to high. Therefor, the IQR based estimators were not considered as good estimators for dealing non-normality.

Finally, we demonstrated the application of GMD as a measure of variability in PCIs ${C}_{pm}$ and ${C}_{pmk}$ for Weibull distribution under low, moderate and high asymmetric conditions. The results indicate that GMD-method works well to some extent under high asymmetry but to get a better estimation of PCIs more research is required.

Beside point estimation, interval estimation of all PCIs was constructed. Moreover, four types of bootstrap confidence intervals i.e., SB, PB, BCPB and PTB and their coverage probabilities using simulation studies were calculated. The selection of the appropriate confidence interval for each method has been made by low average width and higher coverage probability.

The simulations illustrated that ${\text{BCPB}}$ CIs produce the smallest average widths and highest coverage probabilities under all asymmetric levels of Weibull distribution for quantile-based (PC) indices ${C}_{pm}$, and ${C}_{pmk}$. On the other hand, the ${\text{PB}}$ and ${\text{PTB}}$ CIs are recommended for MAD-based indices. Both asymmetric behavior and sample size effect the width and coverage probabilities of confidence intervals. Moreover, coverage probabilities approach to nominal levels with the increase of sample size. The BCPB and ${\text{PB}}$ CIs provides higher coverage probability with a smaller width in case of GMD-based estimators.

## Recommendations

By conducting a comprehensive study, we concluded the following two recommendations.

1. The performance of both modified PCIs is highly effected by asymmetric behavior of the distributions. However, the accurate performance of a particular method for one distribution does not necessitate accurate results for another distribution having different tail behavior.
2. To deal with high asymmetry, more care is needed both for point and interval estimation. In general, in the case of point estimation, quantile-based PC-method leads towards under-estimation, while robust methods like MAD, IQR, and GMD leads towards over-estimation. For interval estimation, a wider spread of CIs was observed under high asymmetry as compared to low and moderate asymmetry.

## Data Availability

The data is given in the paper.
